# Assessment of Fennel Oil Microfluidized Nanoemulsions Stabilization by Advanced Performance Xanthan Gum

**DOI:** 10.3390/foods10040693

**Published:** 2021-03-24

**Authors:** Rubén Llinares, Pablo Ramírez, José Antonio Carmona, Luis Alfonso Trujillo-Cayado, José Muñoz

**Affiliations:** 1 Departamento de Ingeniería Química, Facultad de Química, Universidad de Sevilla c/P, García González, 1, E41012 Sevilla, Spain; rllinares@us.es (R.L.); pramirez@us.es (P.R.); joseacarmona@us.es (J.A.C.); jmunoz@us.es (J.M.); 2 Departamento de Ingeniería Química, Escuela Politécnica Superior, Universidad de Sevilla c/Virgen de África, 7, E41011 Sevilla, Spain

**Keywords:** essential oil, hydrocolloid, microfluidization, nanoemulsion, rheology

## Abstract

In this work, nanoemulsion-based delivery system was developed by encapsulation of fennel essential oil. A response surface methodology was used to study the influence of the processing conditions in order to obtain monomodal nanoemulsions of fennel essential oil using the microchannel homogenization technique. Results showed that it was possible to obtain nanoemulsions with very narrow monomodal distributions that were homogeneous over the whole observation period (three months) when the appropriate mechanical energy was supplied by microfluidization at 14 MPa and 12 passes. Once the optimal processing condition was established, nanoemulsions were formulated with advanced performance xanthan gum, which was used as both viscosity modifier and emulsion stabilizer. As a result, more desirable results with enhanced physical stability and rheological properties were obtained. From the study of mechanical spectra as a function of aging time, the stability of the nanoemulsions weak gels was confirmed. The mechanical spectra as a function of hydrocolloid concentration revealed that the rheological properties are marked by the biopolymer network and could be modulated depending on the amount of added gum. Therefore, this research supports the role of advanced performance xanthan gum as a stabilizer of microfluidized fennel oil-in-water nanoemulsions. In addition, the results of this research could be useful to design and formulate functional oil-in-water nanoemulsions with potential application in the food industry for the delivery of nutraceuticals and antimicrobials.

## 1. Introduction

Essential oils (EOs) are composed of a mixture of volatile hydrophobic compounds produced by aromatic plants which possess interesting properties such as antibacterial, antifungal, antiviral, and antioxidant activity [1,2]. These properties, along with their aromatic properties, give EOs potential applications in a wide variety of fields, such as the food or pharmaceutical industries [2]. Furthermore, their status as safe compounds of biological origin has attracted the attention of researchers and manufacturers. For instance, the use of EOs in food and beverages has risen over the years due to consumer demand for quality and safety in the additives used by the food industry [3]. However, their poor solubility in water, high volatility and undesirable properties when used in high concentrations constrain the direct application of EOs [4,5]. Because of all these limitations, for practical use, they are usually encapsulated as droplets inside an aqueous continuous phase, hence forming emulsions and/or nanoemulsions as a function of the droplet size [6]. The encapsulation of the EOs within the continuous phase prevents the EOs from losing volatiles, avoids oil oxidation and degradation due to environmental stressors, and keeps the biological and physical properties of the EO unchanged [7,8].

Lately, essential oil nanoemulsions have been extensively studied due to their enhanced properties in comparison with conventional emulsions, such as improved stability and the higher bioavailability of lipophilic bioactive compounds [3,9]. These characteristics make nanoemulsions useful tools with great potential in the food sector for the delivery of food ingredients, such as many synthetic and natural antimicrobials, antioxidants, vitamins, flavorings, and colorants [6]. Among all the essential oils studied, several studies have dealt with the formulation and/or application of fennel oil nanoemulsions [10,11,12]. In some of those studies, fennel oil nanoemulsions have shown potential applications as biocides against food borne pathogens [12] and in transdermal drug delivery for antidiabetic action [11].

The addition of a thickener or rheology modifier to previously formed nanoemulsions can both improve long-time stability and expand their applications in fields such as food, pharmaceuticals, and cosmetics. Hydrocolloids have been used for years in food formulations for their outstanding properties as thickening or gelling agents [13]. They may be used to improve the stability and texture of the product and, in addition, for food formulations, modifying the texture will also change the mouth-feel of the emulsion [14]. Furthermore, gelation in emulsions can be achieved by the addition of hydrocolloids which form a network of cross-linked biopolymer molecules where the emulsions droplets are dispersed. Although the mechanical properties of the emulsion with gel-like behavior obtained by this approach are mainly determined by those of the bulk phase biopolymer, the dispersed droplets containing the surfactant may play a strengthening (active filler) or a weakening (inactive filler) role [9,15].

Xanthan gum has been employed as a stabilizer of emulsions and suspensions since it is able to greatly increase the viscosity of aqueous solutions even at low gum concentrations. Furthermore, the highly ordered structure of the rigid molecules of xanthan gum causes the aqueous xanthan gum solution to have interesting viscoelastic properties typical of weak gel systems. This type of rheological behavior is characterized by a mechanical spectrum in which the elastic moduli, G’, are greater than the loss moduli G’’ over the entire frequency range, with the G’’/G’ ratio greater than 0.1. In addition, the flow curves show shear-thinning behavior without yield stress, due to the absence of permanent cross-links, and therefore, weak gels flow but they are not irreversibly broken at large shear rates/deformations [16,17,18]. Advanced performance xanthan gum (APXG) is food-grade xanthan gum (XG) suitable for use in food formulations where fast hydration is required, such as sauces, instant desserts, or beverages. The acetate/pyruvate ratio of conventional XG is 1:1, instead of APXG ratio, which is 10:7. It is stated that the acetate/pyruvate ratio influences the physicochemical and rheological properties of xanthan gum aqueous. It provides high solution viscosity with pseudoplastic properties at low biopolymer concentrations, and presents lower viscous component than the standard xanthan gum, which could be an advantage to stabilize nanoemulsions.

The low water miscibility of several lipophilic bioactive compounds limits their application in aqueous media and their incorporation in food products. Oil-in-water nanoemulsions are in the spotlight of novelty to solve this main problem. The main novelty of this research is the development of stable nanoemulsions formulated with advanced performance xanthan gum and a bioactive volatile oil such as fennel essential oil. For this reason, the present work aims to provide an exhaustive study of the influence of the formulation and processing on microfluidized nanoemulsion properties. In this work, a xanthan gum with boosted rheological properties (advance performance xanthan) e 0.1–0.4/100 g [19,20,21]. The goal of the present study is to obtain the optimum processing conditions for achieving fennel oil nanoemulsions with the lowest droplet size and narrowest distribution and study the influence of the addition of different concentrations of a hydrocolloid (xanthan gum advanced performance) to the stability and mechanical properties of the previously formed nanoemulsions.

## 2. Materials and Methods

### 2.1. Materials

Sweet fennel essential oil (ρ = 0.933 g·cm^−3^) was provided by Sigma-Aldrich and was used as the dispersed phase in the O/W nanoemulsions. Tween 80 (ρ = 1.064 g·cm^−3^ and HLB = 15) was provided by Sigma-Aldrich and was used as emulsifier. Food-grade xanthan gum “Advanced Performance” was provided by C.P. Kelco (Atlanta, GA, USA).

### 2.2. Chromatographic Analysis

Samples for GC-MS analysis were prepared by dissolving 100 μL of essential oil in 1 mL of dichloromethane. The chromatographic analysis of fennel oil was carried out following the procedure of Miraldi [22] on a Trace 1300 (Thermo Fisher, Waltham, MA, USA) gas chromatograph to a TSQ 8000 (Thermo Fisher, Waltham, MA, USA) triple quadrupole mass spectrometer. A Zebron ZB-WAXplus column (30 m, 0.25 mm, 0.25 mm) and helium as carrier gas (flow rate 1.0 mL/min) were used. Samples were injected using the split sampling technique, ratio 1:45; sample amount injected was 0.5 mL; injection port temperature, 250 °C. Oven temperature was held at 30 °C for 8 min, then programmed at 30 °C/min to 180 °C, held there for 5 min, and then again programmed at 10 °C/min to 220 °C and held for 10 min. The MS operating parameters were: electron ionization, 70 eV; accelerating voltage, 8 kV; scan range, 30 ± 650 amu. Identification and quantification of the volatile compounds was carried out by a peak-matching library search using the NBS/NIST 11 library.

Table 1 shows the results obtained from the chromatogram of fennel essential oil. The composition of this natural oil is complex since it contains several compounds (up to 12). This composition is similar to other fennel essential oils reported by different authors [23,24,25]. However, this fennel oil presents a major concentration of anethole. Anethole is a widely used flavoring substance with antimicrobial and antifungal activity [26]. D-limonene was the second major component detected in this fennel oil, followed by fenchone and α-pinene. According to Bilia et al., fenchone concentration in fennel essential oil should be less than 7.5% [27].

### 2.3. Emulsification Process

The emulsification process was performed in two steps. First, a coarse emulsion (200 g) was obtained using a rotor-stator system Silverson L5M (Silverson, East Longmeadow, MA, USA) at 6000 rpm for 30 s. Secondly, the coarse emulsion, previously cooled down to 5 °C, was processed in a high energy homogenization device Microfluidizer M-110P (Microfluidics, Westwood, MA, USA) with two interaction chambers (F12Y and H30Z). The use of two interaction chambers allows better droplet size distributions to be obtained [28]. All nanoemulsions contained 1 g/100 g of EO and a surfactant-to-oil ratio 3:1. The selection of the formulation is based on previous studies so that it results in stable nanoemulsions.

### 2.4. Gum Addition

The advanced performance xanthan gum was directly added into the previously formed nanoemulsions and gently stirred until complete dissolution. Different samples with a total concentration of 0.05, 0.10, 0.25, 0.40, 0.55, and 0.70 g/100 g were prepared. Samples were stored at 4 °C for 24 h prior to measurement.

### 2.5. Size Measurement

The droplet size distribution was obtained by using a Zetasizer^®^ ZS (Malvern, Worcestershire, UK). The droplet size of the emulsions was described as the mean diameter (Z-average), whereas the width of the size distribution was indicated by the polydispersity index (PdI). PdI is a measure of the heterogeneity of a sample based on size. This index is a dimensionless number that is calculated from a two-parameter fit to the correlation data. Polydispersity index values smaller than 0.05 are mainly seen with highly monodisperse standards. The refractive index values were 1.538, and 1.343 for the oil phase and the continuous medium, respectively. All measurements were made in triplicate.

### 2.6. Oscillatory and Flow Curve Measurement

The rheological properties were measured by a controlled-stress AR2000 rheometer (TA Instrument, New Castle, DE, USA) using a serrated plate-plate geometry (60 mm diameter; gap = 1 mm) to avoid slippage of the sample.

Shear stress sweep tests were carried out at a constant frequency of 1 Hz from 0.01 to 50 Pa. The frequency sweep was then carried out at a constant stress value within the linear viscoelastic region in the frequency range from 20 to 0.10 rad/s. The flow curves were measured from 0.01 to 35 Pa, following a step-wise protocol with a maximum time per point of 3 min.

All rheological measurements were repeated at least three times at a controlled temperature of 20 °C and using a solvent trap to prevent solvent evaporation.

### 2.7. Microstructure of the Emulsions and Continuous Phases

In order to observe the differences between the microstructure of the emulsions and the continuous phases, selected samples were characterized by Cryo-scanning electronic microscopy (Cryo-SEM). A scanning electron microscope Zeiss EVO SEM (Zeiss, Oberkochen, Germany) was used following the procedure described by Carmona et al. [29].

### 2.8. Statistical Analysis

Experimental data of the study of the influence of the homogenization pressure and number of cycles on nanoemulsion droplet size distributions were fitted to the following quadratic model:

Y = β_0_ + β_1_·P + β_2_·N + β_12_·P·N + β_11_·P^2^ + β_22_·N^2^, (1)

where Y is the response variable (dependent variable), β_0_ is the constant, and P (homogenization pressure) and N (number of cycles) are the coded independent factors. The dependent variables considered were the Z-average and the polydispersity index (PdI) for nanoemulsions aged for 1 day. Statistical analysis of the data was performed with Statistic 10.0 software (StatSoft Inc., Tulsa, OK, USA) to evaluate the analysis of variance (ANOVA). For model construction, terms with *p* > 0.05 were removed and the analysis was recalculated without these terms. The suitability of the models was determined by using the coefficient of determination. All statistical calculations were conducted at a significance level of *p* = 0.05.

## 3. Results and Discussion

### 3.1. Influence of Energy Input on Droplet Size Distribution

**Figure 1:**
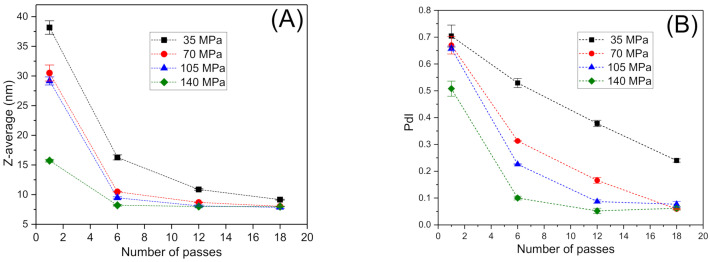
(**A**) Droplet mean diameters and (**B**) polydispersity index values for emulsions aged for 24 h as a function of the number of passes and homogenization pressure. Vertical bars indicate standard deviation of the mean (three replicates).

Energy input in a microfluidizer device is a function of homogenization pressure (P) and the number of cycles (N). Hence, the influence of these variables on droplet size distribution (DSD) for essential oil nanoemulsions has been studied in several works [30,31]. It has been observed that increasing homogenization pressure and the number of cycles reduces the mean droplet sizes and polydispersity of emulsions until a plateau value of both parameters is obtained. Two main variables are commonly used to characterize DSD for Zetasizer devices: Z-average and the polydispersity index (PdI). Figure 1A,B show Z-average and PdI values as a function of P and N, respectively. A clear tendency to decreasing droplet sizes (Z-average) with an increase in homogenization pressure and the number of cycles was observed. It is important to note that nanoemulsions prepared using 12 or more cycles at the highest homogenization pressures showed mean diameters below 10 nm. Nevertheless, results of the ANOVA test demonstrated that there are not significant differences in Z-average for emulsions aged for 24 h prepared in the 12–18 passes range at 140 MPa. Low droplet mean diameters could be a great advantage against some destabilization mechanism, i.e., coalescence and creaming [32]. The lowest value of PdI was obtained for the nanoemulsion processed with 12 passes through the microfluidizer at 140 MPa. However, results of ANOVA test demonstrated that the number of passes in the 12–18 range had no significant effect upon the PdI of the nanoemulsions.

**Figure 2:**
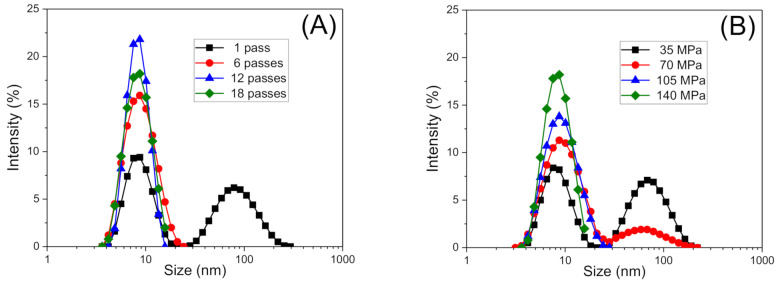
Droplet size distributions for emulsions aged for 24 h as a function of (**A**) the number of passes at a fixed homogenization pressure of 140 MPa and (**B**) homogenization pressure processed at a fixed number of passes of 18.

Figure 2A shows, by way of example, the influence of the number of passes on the droplet size distributions of the nanoemulsions aged for 24 h and processed at a fixed homogenization pressure of 140 MPa. All emulsions in the 6–18 passes range showed monomodal distributions. Conversely, the nanoemulsion developed using only one cycle exhibits a very wide distribution with two peaks centered around 8 and 80 nm, which is probably due to the lack of mechanical energy input during the emulsification process. This fact is also presented in the decrease in polydispersity for nanoemulsions developed at pressure in the range 105–140 MPa comparing with those developed at lower homogenization pressures (see Figure 2B). When the nanoemulsion passed through the high-pressure homogenizer microchannels at higher homogenization pressures, the droplet size distributions become monomodal.

**Figure 3:**
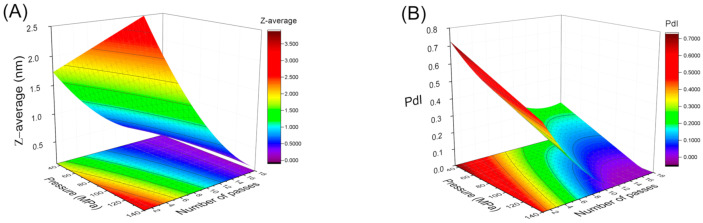
Response surface 3D plot of (**A**) droplet mean diameter and (**B**) polydispersity index as a function of the number of passes and homogenization pressure for emulsions aged for 24 h.

Figure 3A,B illustrates the three-dimension response surface curve of Z-average and polydispersity index for the studied variables (homogenization pressures and number of cycles). This graph provides a visual interpretation of the interaction between the factors analyzed. In addition, response surface methodology is a very powerful tool for the development of new systems. This methodology can link the processing variables with response variables in order to obtain a mathematical model that fits the behavior of nanoemulsions. Z-average fitted a quadratic function of homogenization pressure (P) and number of passes (N):

Z-average = 8.14 − 4.00·P − 8.99·N + 4.59·P·N + 10.02·N^2^. (2)

The results indicated that the model employed was adequate, showing a very satisfactory value of *R*^2^ = 0.917 and no significant lack of fit (F_crit_ > F_lof_, with *p* = 0.05). The model predicted that Z-average was sensitive to all studied variables. The most significant factor affecting this parameter was the number of passes (N), as supported by their linear and quadratic coefficients (−8.99 and 10.02):

PdI = 0.18 − 0.13·P − 0.25·N + 0.17·N^2^. (3)

The coefficient of determination (*R*^2^) value of 0.933 indicated a good correlation between the experimental results and predicted responses. It is worth noting the existence of a minimum value of the polydispersity index for the highest value of the number of cycles (N) and homogenization pressure (P). From the model we deduced that the most significant factors affecting PdI in these fennel oil nanoemulsions were the linear term of the homogenization pressure and the linear and quadratic terms of the number of passes.

An optimum process condition can be set for emulsions with minimum droplet sizes and polydispersity. According to the response surface analysis, the predicted minimum Z average mean diameter and polydispersity value are obtained when P = 140 MPa and N = 12 passes. For this reason, these conditions were fixed for the following tests. In this study, minimum initial droplet sizes were observed for all formulations in comparison to those obtained in other studies in which fennel oil nanoemulsions are developed [10].

### 3.2. Rheological Behavior Influence of Xanthan Gum Concentration

First, the viscosity of fennel oil nanoemulsion with processing conditions of 140 MPa and 12 cycles was studied. All systems presented a Newtonian flow behavior with a viscosity of 1.63 ± 0.05 mPa at 20 °C (data not shown). In order to expand the application of previously obtained nanoemulsions, a natural hydrocolloid (xanthan gum) was added. In this way, the mechanical properties of previously prepared nanoemulsions can be modified as a function of xanthan gum concentration, thus obtaining nanoemulsion gels of tailored viscoelasticity. The rheological properties were studied for xanthan gum concentrations ranging from 0.05 to 0.70 g/100 g.

**Figure 4:**
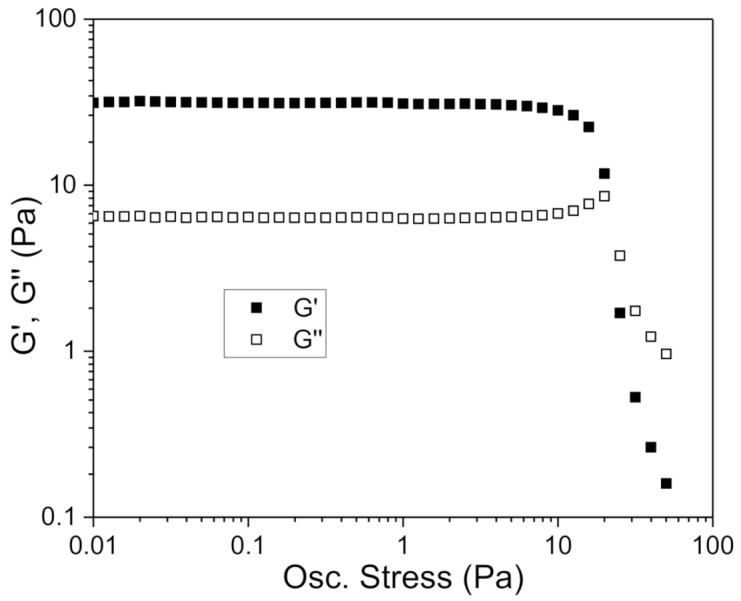
Storage (G’) and loss (G’’) moduli at 1 Hz as a function of shear stress for the fennel oil nanoemulsion with 0.70 g/100 g of the AP xanthan gum. Standard deviation of the mean (three replicates) for all the data are below 5%. (T = 20 °C).

Most applications of oscillatory tests entail working in linear viscoelastic conditions, that is, in non-destructive conditions. Therefore, a prior step for the determination of viscoelastic properties by this technique consists of selecting a shear stress value that guarantees that the structure of the material is not irreversibly destroyed in the frequency range in which the test is performed. The determination of the linear viscoelastic range was carried out from stress sweep tests, at a fixed frequency of 1 Hz. The critical shear stresses for each concentration were determined as the shear stress value from which the G’ modulus varied more than 2% with respect to the average G’ values in the linear region. G’ values have been used to obtain the shear stress value instead of G’’ due to the fact that a departure from a constant value was observed at lower shear stresses for storage moduli for all the concentrations studied. Figure 4 shows the shear stress sweep test for the sample with a xanthan gum concentration of 0.70 g/100 g as an example.

**Figure 5:**
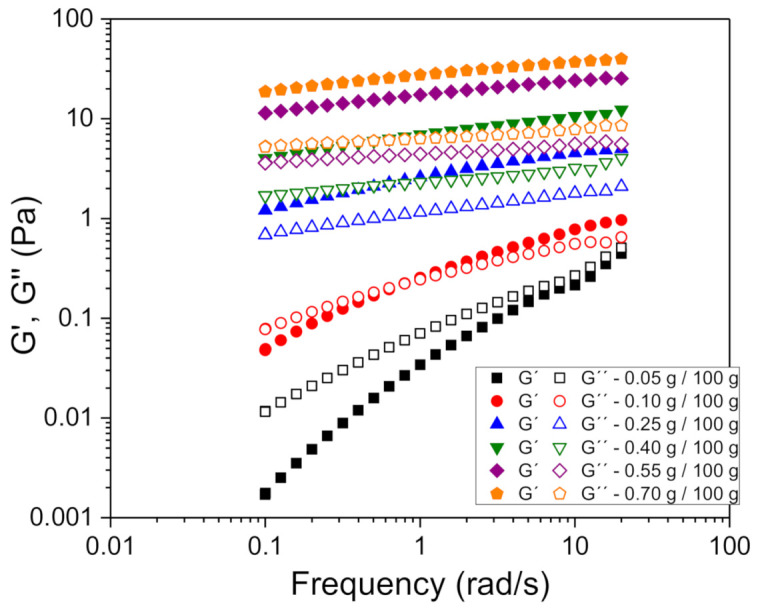
Mechanical spectra for fennel oil nanoemulsions with different xanthan gum concentration at 20 °C. Data shown are the average values of three replicates. The standard deviation for all the experiments was lower than 5%.

Small amplitude oscillatory shear measurements were carried out as shown in Figure 5. Two different viscoelastic behaviors were clearly observed. For xanthan gum concentrations below 0.25 wt.%, a crossover frequency which shifts towards higher values was observed. This crossover is associated with a change of viscoelastic behavior from liquid-like to gel-like. This behavior is typical for diluted solutions of hydrocolloids with low interactions between the polymer chains [33,34]. Nevertheless, for xanthan concentration of 0.25 wt.% and above, the mechanical spectra obtained are completely different. For those systems, the storage modulus (G’) was above the loss modulus (G’’) for the whole frequency range, which indicates the occurrence of a more compact structure typical of systems which exhibit weak gel-like behavior [34].

With increasing the gum concentration, the system shows a higher weak gel character, with higher values of G’ and G”, increasing differences between G’ and G” and a reduction in the slope of G’. Thus, the slope of G’ can be used to quantify the solid-like behavior of the system [35]. A marked decrease in the G’ slope is observed with increasing xanthan gum concentration. Comparison of the values of the mechanical spectra from the gelled nanoemulsions with the mechanical spectra of the same xanthan gum network in aqueous solution shows similar value [20]. Hence, it is confirmed that the rheological properties of the weak gelled nanoemulsions are dominated by the biopolymer network which controls the structure of the continuous phase.

**Figure 6:**
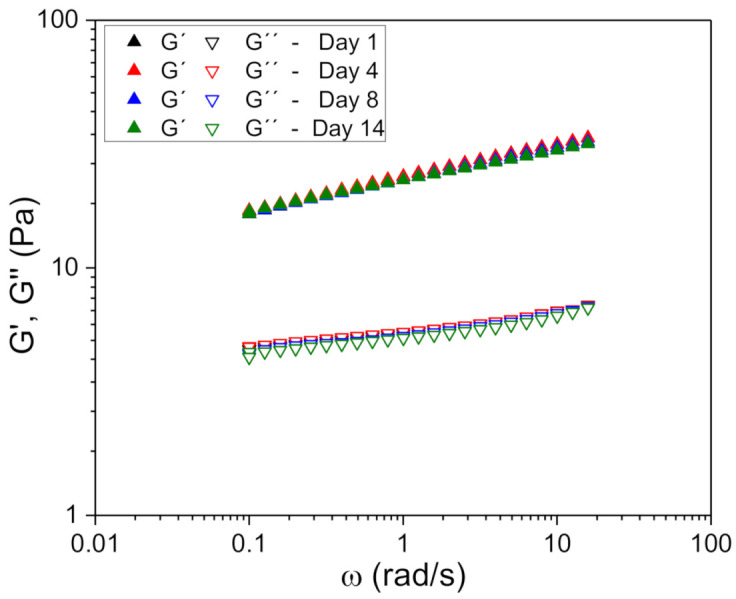
Mechanical spectra as a function of aging time for fennel oil nanoemulsion formulated with advanced performance xanthan gum (0.70 g/100 g) at 20 °C. Data shown are the average values of three replicates. The standard deviation of for all the experiments was lower than 5%.

When working in the linear viscoelastic domain, the test can be considered non-destructive. Therefore, the results obtained can be related to the structure of the sample and are very sensitive to changes in it [3]. Hence, obtaining mechanical spectra as a function of the storage time of the samples gives us reliable information concerning the possible presence of destabilization processes that cause changes in the structure of the sample. In Figure 6, the evolution over time of the mechanical spectra for the sample of 0.70% by weight of xanthan gum is shown as an example. It was observed that for all the studied concentrations the variations in the values of the viscoelastic modules with respect to the replicates and time of aging were less than 5%.

**Figure 7:**
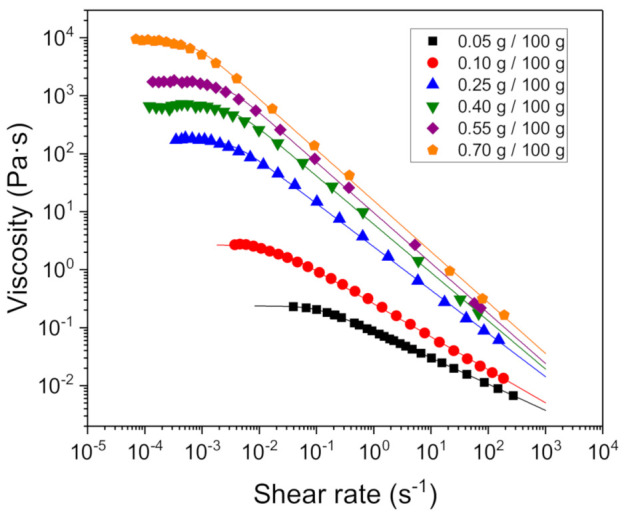
Steady state viscosity as a function of shear rate for the fennel oil nanoemulsion with different AP xanthan gum concentrations at 20 °C. The lines are the best fit to the Carreau model, whose parameters are given in Table 2. Data shown are the average value of three replicates with a standard deviation for all the experiments lower than 10%.

In order to fulfil the rheological characterization of the gels containing the fennel oil nanoemulsions, flow curves were carried out as a function of the xanthan gum concentration (Figure 7).

It is observed that all the systems showed shear-thinning behavior characterized by a constant viscosity at low shear rates values (the zero-shear viscosity, *η*_0_) which follows a power-law decay for shear rates below a critical value ($\dot{\gamma_{c}}$). Therefore, the experimental viscosities (*η*) can be fitted to the well-known Carreau model:

(4) $$\eta =\frac{\eta_{0}}{{\left(1+{\left(\frac{\dot{\gamma }}{{\dot{\gamma }}_{c}}\right)}^{2}\right)}^{\left(\frac{1-n}{2}\right)}}$$

where *n* is the flow index. Solid lines indicating the best fit to the experimental data with the parameter values are given in Table 2. A good agreement between experimental data and the estimated values from the model is obtained. The zero shear viscosity values were compared with those obtained for the same hydrocolloid in aqueous solution [21]. It was observed that for the same concentration, the *η*_0_ values were lower for the nanoemulsion systems (see Table 3). These changes in the zero shear viscosity values between the nanoemulsions and the aqueous solutions of advanced performance xanthan gum are likely to be related to microstructural changes.

**Figure 8:**
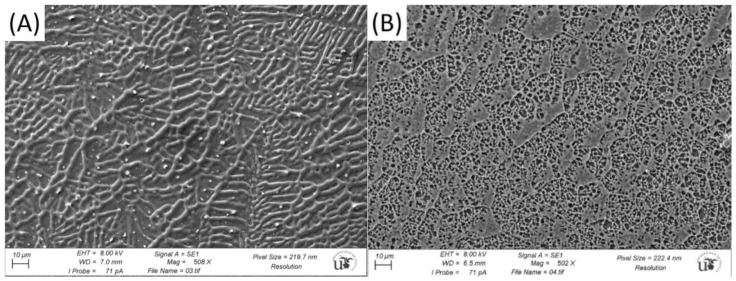
Representative Cryo-SEM micrographs for (**A**) the emulsion formulated with 0.25 g/100 g of advanced performance xanthan gum and (**B**) an aqueous solution that contains 0.25 g/100 g of advanced performance xanthan gum.

Therefore, in order to establish a relationship between rheological performance and microstructural conformation, cryo-SEM images of an emulsion that contains an advanced performance xanthan gum concentration of 0.25 g/100 g and the correspondent aqueous solutions were obtained (Figure 8A and Figure 8B, respectively). A more structured system is observed in Figure 8B, which corresponds to the aqueous solutions. This structural modification is likely to be related to the fact that nanoemulsion droplets behave as an inactive filler, leading to a slight decrease in the biopolymer network (Figure 8A).

## 4. Conclusions

Stable edible nanoemulsions containing sweet fennel essential oil with tween 80 as emulsifier were processed using a two-step emulsification consisting of a rotor/stator pre-homogenization and microfluidic homogenization. Narrow monomodal distributions (PdI ≈ 0.05) with low average size (Z-average ≈ 8 nm) were obtained and the effect of processing variables on the final nanoemulsion was studied, establishing optimum processing conditions. Xanthan gum was added to the nanoemulsion as a thickening agent to improve the rheological properties. The systems showed viscoelastic properties for every gum concentration used and were characterized by small amplitude oscillatory shear tests and steady flow measurements. Edible nanoemulsion weak gels were obtained with the addition of xanthan gum concentrations above 0.25 g/100 g. The gel strength and zero shear viscosity were functions of the concentration of the gum and, therefore can be conveniently adjusted. Furthermore, from the steadiness of the mechanical spectra over time, the system proved to be stable in the xanthan gum concentration range studied. Comparison of the rheological properties and microstructure of the nanoemulsion weak gels with the ones of the same concentration of xanthan gum aqueous solutions revealed that the mechanical properties are governed by the biopolymer network and the nanoemulsion droplets behave as inactive fillers, slightly decreasing the zero-shear viscosity of the biopolymer network.

## Figures and Tables

**Table 1:** Percentage composition of the fennel essential oil used in the present study.

Compound	Relative Concentration (%)
α-Pinene	6.8
β-Pinene	0.3
β-Phellandrene	0.6
α-Phellandrene	1.2
β-Myrcene	0.8
D-Limonene	16.7
p-Cymene	0.7
Fenchone	7.4
Estragole	2.3
Anethole	60.6
p-Anisaldehyde	2.3
p-Acetonylanisole	0.3

**Table 2:** Fitting parameter values for the Carreau model. The values were obtained by a non-linear regression analysis.

[XG] (g/100 g)	*η*_0_ (Pa·s)	${\dot{\gamma }}_{c}$ (s^−1^)	*n*
0.05	0.24 ± 0.01	0.11 ± 0.02	0.55 ± 0.01
0.10	2.7 ± 0.1	0.016 ± 0.002	0.43 ± 0.01
0.25	175 ± 9	3.3 × 10^−3^ ± 4 × 10^−4^	0.25 ± 0.01
0.40	660 ± 28	3.6 × 10^−3^ ± 5 × 10^−4^	0.17 ± 0.02
0.55	1730 ± 80	2.5 × 10^−3^ ± 3 × 10^−4^	0.13 ± 0.01
0.70	8900 ± 508	7 × 10^−4^ ± 1 × 10^−4^	0.12 ± 0.01

**Table 3:** Comparison of the zero shear viscosity values of the weak gel advanced performance xanthan gum systems containing fennel oil nanoemulsion (* this work) or without fennel oil nanoemulsion (**, Reference [21] by Carmona et al.).

[XG] (g/100 g)	*η*_0_ (Pa·s) *	*η*_0_ (Pa·s) **
0.25	175 ± 9	214 ± 5
0.40	660 ± 28	1638 ± 15

## Data Availability

The data presented in this study are available on request from the corresponding author.
